# Comedogenic lupus: a rare variant of chronic cutaneous lupus erythematosus – case series^⋆^

**DOI:** 10.1016/j.abd.2022.04.003

**Published:** 2023-01-25

**Authors:** Lucas Campos Garcia, Isabela Boechat Morato, Raquel Ferreira Queiroz de Melo, Everton Carlos Siviero do Vale

**Affiliations:** a Dermatology Service, Hospital das Clínicas, Universidade Federal de Minas Gerais, Belo Horizonte, MG, Brazil; b Department of Internal Medicine, Faculty of Medicine, Universidade Federal de Minas Gerais, Belo Horizonte, MG, Brazil

**Keywords:** Acneiform eruptions, Case reports, Lupus erythematosus, cutaneous, Lupus erythematosus, discoid, Lupus erythematosus, systemic

## Abstract

**Background:**

Comedogenic lupus is an uncommon variant of cutaneous lupus, clinically characterized by the presence of comedones, papules and erythematous-infiltrated plaques, cysts and scars in photo-exposed areas, mimicking acne vulgaris and acneiform eruptions.

**Objectives:**

To report clinicopathological characteristics of patients with comedogenic lupus in a tertiary dermatology service over a 15-year period and review cases described in the literature.

**Methods:**

Retrospective study of patients with clinical and histopathological diagnoses of comedogenic lupus between the years 2006 and 2021. The literature search was carried out in the PubMed and VHL Regional Portal databases, using the terms: “comedogenic lupus” and “acneiform lupus” in Portuguese and English.

**Results:**

Five patients were diagnosed during the described period, all female, with a mean age of 56.6 years. Smoking was observed in three cases, as well as pruritus. The most affected site was the face, especially the pre-auricular, malar and chin regions. Follicular plugs, epidermal thinning and liquefaction degeneration of the basal layer were predominant histopathological findings. Hydroxychloroquine was used as the first-line treatment; however, other medications were used, such as dapsone, methotrexate, tretinoin cream, and topical corticosteroids. The literature search identified 17 cases, with a mean age of 38.9 years, 82% of which were women. Only 23% had a diagnosis of systemic lupus erythematosus. Hydroxychloroquine was the most recommended systemic medication.

**Study limitations:**

Retrospective, single-center study. The literature search was carried out in two databases.

**Conclusions:**

Dermatologists should be aware of acneiform conditions with poor response to the usual treatment. Early diagnosis and treatment reduce the risk of unaesthetic scars.

## Introduction

Chronic cutaneous lupus erythematosus (CCLE) has more than twenty described clinical variants.^1^ Some of these variants, such as discoid lupus erythematosus (DLE), the most common chronic subtype, exhibit striking clinical features that contribute to diagnostic suspicion. Other variants, however, such as comedogenic lupus (CL), can pose a diagnostic challenge.^1^ CL is a rare form of CCLE,^1^ which is clinically characterized by multiple erythematous papules, comedones, cysts, and acneiform scars in sun-exposed areas, which may be associated with typical DLE lesions.^2^ Although uncommon, CL should be considered in the differential diagnosis of acne vulgaris and acneiform eruptions refractory to conventional treatment.^1^ The present study reports five cases of CL and compares their demographic data, clinical characteristics and treatment with those of the 17 published cases, in addition to illustrating the different types of skin lesions and discussing the main histopathological findings.

## Methods

This is a single-center, retrospective study carried out at the Dermatology Service of Hospital das Clínicas, Universidade Federal de Minas Gerais. Data from patients diagnosed with CL in the last 15 years, between 2006 and 2021, were collected. The literature search was carried out using PubMed and VHL (Virtual Health Library) Regional Portal databases using the terms “comedonal lupus” and “acneiform lupus” in Portuguese and English. Ten articles were retrieved by this search, while another six were identified from the bibliographic references.

## Results

During the study period, from 2006 to 2021, five cases diagnosed as CL were identified, which are described below. The literature search identified 17 cases of the comedogenic variant. The main clinical and demographic characteristics of the cases in this series and those reported in the literature are described in Table 1^3^, ^4^, ^5^, ^6^, ^7^, ^8^, ^9^, ^10^, ^11^, ^12^, ^13^, ^14^, ^15^, ^16^ and the histopathological findings of the five cases in this series are listed in Table 2.

**Figure 1:**
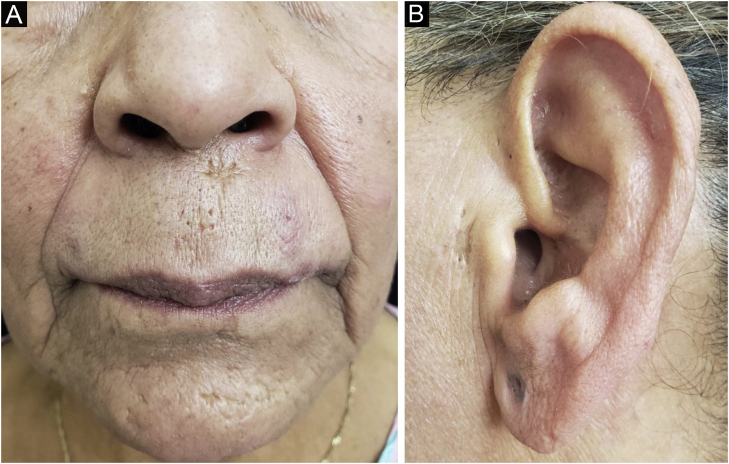
Open comedones and acneiform scars on the supralabial, mentum and preauricular regions after treatment.

Case 1 A 45-year-old female patient, non-smoker, had been undergoing treatment for acne vulgaris for years, with unsatisfactory response. On examination, she had erythematous papules, open comedones, and cysts on the submandibular, chin and supralabial regions (Fig. 1). She had had a diagnosis of DLE 18 years ago, with dyschromic atrophic plaques on the face and scalp. A biopsy of the area with cysts and comedones was performed and the histopathological findings confirmed the diagnosis of CL (Table 2). Methotrexate 15 mg/week, dapsone 100 mg/day, and betamethasone dipropionate ointment were prescribed and surgical excision of the cystic lesions was performed, with some punctate scars remaining. She did not meet clinical or laboratory criteria for systemic lupus erythematosus (SLE).

**Figure 2:**
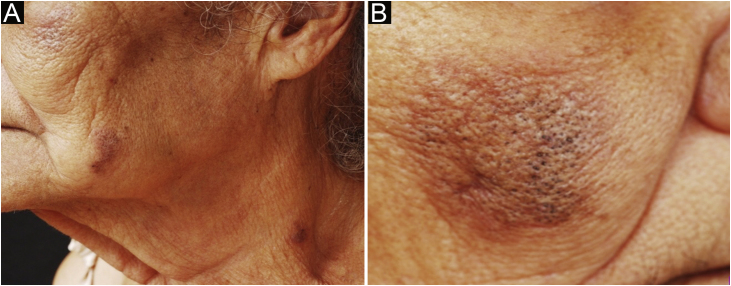
(A–B) Infiltrated erythematous plaques, containing open comedones, on the malar, mandibular and cervical regions.

Case 2 An 85-year-old female patient, non-smoker, presented open comedones on erythematous, infiltrated, and pruritic plaques on the mandibular, malar and cervical regions for the last year (Fig. 2). The scalp showed extensive areas of alopecia with dyschromia, scaling, and follicular keratosis. The anatomopathological examination of a malar region skin specimen was compatible with CL (Table 2). She had a history of pericardial effusion, undergoing etiological investigation. The subsequent laboratory examination showed proteinuria, reduced glomerular filtration rate, presence of urinary cell casts, complement consumption, antinuclear antibody (ANA) 1:640, with a coarse speckled nuclear pattern, and SLE was diagnosed. After three months of treatment with hydroxychloroquine 400 mg/day, systemic corticosteroid therapy, and photoprotection, there was partial improvement of the acneiform lesions, resolution of pruritus and control of the systemic disease activity.

**Figure 3:**
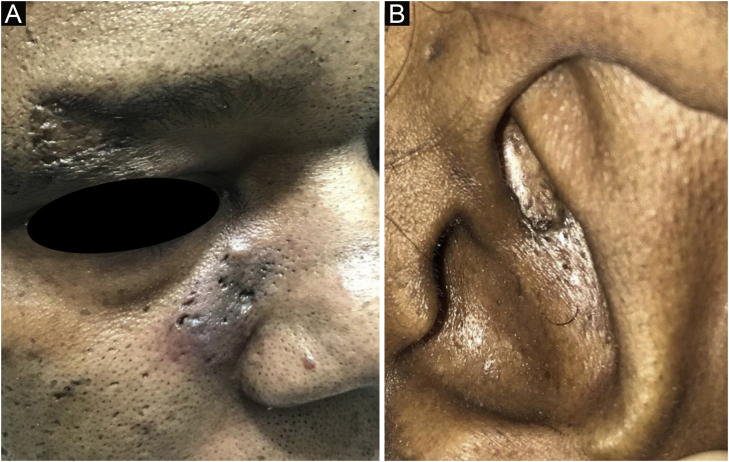
Erythematous and hyperchromic plaque with comedones on the paranasal, malar, supraorbital (A) and auricular (B) regions.

Case 3 A 41-year-old female patient, smoker, presented with infiltrated erythematous plaques for two years, containing open comedones, punctate scars and cysts in the auricular, paranasal, and malar regions (Fig. 3). She reported pruritus and pain. The anatomopathological examination was compatible with CL (Table 2). She also had erythematous-dyschromic discoid plaques on the arms, clinically compatible with DLE. In the subsequent laboratory examination, she had ANA 1: 160, with a dense fine speckled nuclear pattern, without any other criteria for SLE. Systemic therapy was implemented with hydroxychloroquine 400 mg/day, methotrexate 15 mg/week and intralesional corticosteroids, with partial improvement of the lesions after eight months.

**Figure 4:**
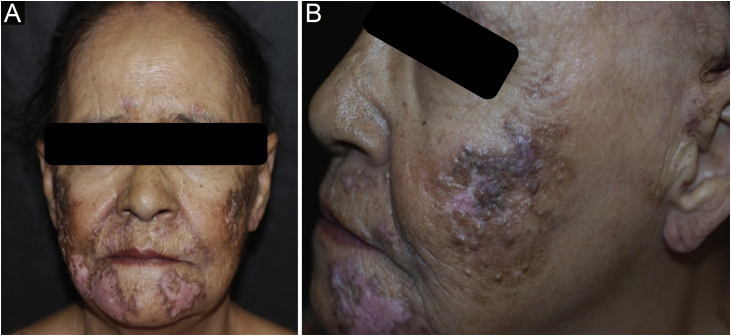
Open and closed comedones over active discoid lupus erythematosus plaques; (A) Distribution of plaques on the face; (B) Detail of the lesion.

**Figure 5:**
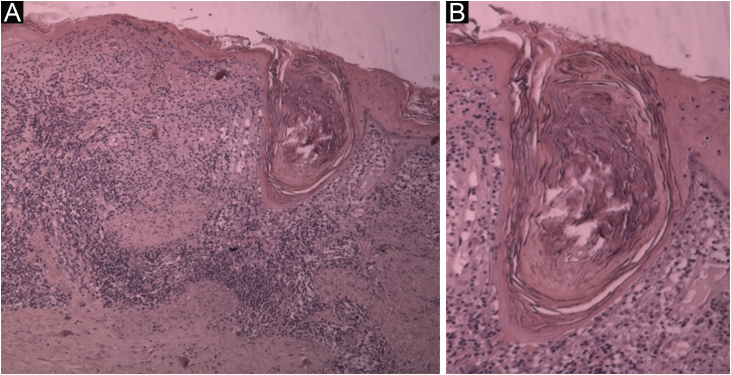
(A) Histological section showing hyperkeratosis, epidermal thinning, liquefaction degeneration of the basal layer and large follicular plug; in the dermis, superficial and deep periadnexal and perivascular mononuclear infiltrate (Hematoxylin & eosin, ×40). (B) Detail of the follicular plug obstructing a dilated hair follicle. Note the liquefaction degeneration of the follicular wall and adjacent epidermis (Hematoxylin & eosin, ×100).

Case 4 A 50-year-old female patient, hypertensive and a smoker, diagnosed with DLE for three years, was treated with chloroquine diphosphate 250 mg/day. She developed multiple open and closed comedones on previous DLE plaques on the nasal, pre-auricular, malar and mentum regions (Fig. 4). The anatomopathological examination of the preauricular lesion was compatible with CL (Fig. 5 and Table 2). ANA was negative, without clinical and laboratory criteria for SLE. During the follow-up, 15 mg/day of methotrexate was added to the treatment, in addition to tretinoin cream 0.025% and benzoyl peroxide 5%. After one year of follow-up, chloroquine diphosphate was replaced by dapsone 100 mg/day, due to bilateral macules, but the patient did not tolerate the medication. There was a partial improvement after the introduction of doxycycline 200 mg/day.

**Figure 6:**
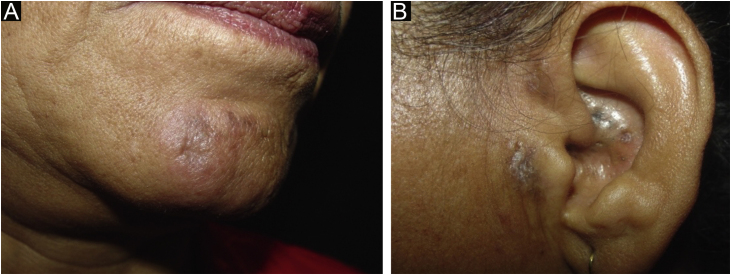
(A) Comedones and depressed scar over erythematous plaque on the mentum. (B) Preauricular discoid lupus erythematosus plaque and open comedones on the auricular region.

Case 5 A 62-year-old female patient, smoker, diagnosed with DLE for fifteen years, had plaques with open and closed comedones on the mentum, preauricular and auricle regions (Fig. 6). The anatomopathological examination of the preauricular lesion was compatible with CL (Table 2). ANA was negative, without clinical and laboratory criteria for SLE. She has received betamethasone dipropionate and tretinoin cream 0.025%, with good control.

**Table 1:** Main clinical and demographic characteristics of the cases in the present series and of the 17 series published in the literature.

Case	Year of Publication	Age	Sex	Smoker	Time of evolution	Pruritus	Sites	ANA	SLE	DLE	Prior diagnosis of acne	Treatment	Evolution
Case 1		45	F	No	18 years	No	Face (submandibular, mentum, supralabial)	Negative	No	Yes	Yes	Methotrexate 15 mg/week, dapsone 100 mg/day, betamethasone dipropionate ointment, surgical excision (cysts)	Improvement
Case 2		85	F	No	1 year	Yes	Face (mandibular, malar) and cervical regions	1:640 CSN	Yes	Yes	No	Hydroxychloroquine 400 mg/day, systemic corticosteroid therapy, photoprotection	Partial improvement
Case 3		41	F	Yes	2 years	Yes	Face (paranasal, maxillae) and auricle	1:160 FSN	No	Yes	No	Hydroxychloroquine 400 mg/day, methotrexate 15 mg/week, intralesional corticosteroid, photoprotection	Partial improvement
Case 4		50	F	Yes	2 years	Yes	Face (nasal, pre-auricular, malar, mentum)	Negative	No	Yes	No	Methotrexate 15 mg/week, dapsone 100 mg/day, tretinoin cream 0.025%, benzoyl peroxide 5%, doxycycline 200 mg/day	Partial improvement
Case 5		62	F	Yes	15 years	No	Face (mentum, pre-auricular) and auricular region	Negative	No	Yes	No	Topical corticosteroids, tretinoin cream 0.025%	Improvement
Haroon et al.^3^	1972	32	F	No	4 years	Yes	Back	Negative	No	Yes	No	Not informed	Not reported
Motel et al.^4^	1995	24	F	No	Not informed	No	Face and cervical region	1:5120 Nucleolar	Yes	No	Yes	Erythromycin 250 mg QID	No improvement
Motel et al.^4^	1995	29	F	No	7 years	Yes	Face and upper trunk	1:2560 HN	Yes	No	Yes	Tetracycline and erythromycin	No improvement
Chang et al.^5^	2006	32	F	No	3 years	No	Face (nasolabial fold)	Negative	No	Yes	No	Not reported	Not reported
El Sayed et al.^6^	2007	60	M	No	8 years	Yes	Face (malar)	1:80 FSN	No	No	No	Hydroxychloroquine 400 mg/day, photoprotection, topical clobetasol and tazarotene	Improvement
Stavrakoglou et al.^2^	2008	38	M	No	7 years	Yes	Face, back and anterior thorax	Negative	No	No	Yes	Hydroxychloroquine 400 mg/day, photoprotection	Complete improvement in 1 year
Hemmati et al.^7^	2009	33	F	No	1,5 years	No	Scalp	Not informed	No	Yes	Yes	Hydroxychloroquine 400 mg/day, photoprotection, triamcinolone infiltration, manual extraction	Improvement
Farias et al.^8^	2011	35	F	No	2 years	Yes	Face (nasal dorsum, mentum) and ears	Not informed	No	Yes	Yes	Hydroxychloroquine 400 mg/day, tetracycline, photoprotection	Improvement in 6 months
Ugarte et al.^9^	2014	41	F	‒	11 years	No	Face, pre-auricular and scalp regions	1:320^a^	No	No	‒	Hydroxychloroquine 400 mg/day and prednisone 20 mg/day	Remission
Deruelle-Khazaal et al.^10^	2017	32	F	No	2 years	Yes	Face (mentum) and ear	Positive^b^	No	Yes	Yes	Prednisone and chloroquine diphosphate	Pruritus improvement
Vieira et al.^11^	2018	32	F	No	2 years	Yes	Face (mentum)	Negative	No	Yes	Yes	Prednisone and chloroquine diphosphate	Improvement in 3 months
Mohanty et al.^12^	2019	20	F	No	8 months	No	Face, cervical, dorsum and abdomen	Positive^b^	Yes	No	Yes	Methotrexate 15 mg/week, and hydroxychloroquine 200 mg/day	Not reported
Droesch et al.^1^	2019	57	F	No	1 year	No	Face (malar, mentum, forehead) and cervical regions	1:80 speckled nuclear	No	Yes	Yes	Hydroxychloroquine 200 mg/day	Lost to follow-up
Zhou et al.^13^	2019	54	M	No	2 years	No	Scalp	Negative	No	Yes	Yes	Oral hydroxychloroquine and topical isotretinoin 0.01%	Reduction of comedones in 3 months
Cozzani et al.^14^	2020	45	F	No	1 year	Yes	Ears	Negative	No	Yes	No	Photoprotection	Stability
Gaitibi et al.^15^	2021	50	F	‒	1 year	No	Scalp	Negative	No	No	No	Not reported	Not reported
Chessé et al.^16^	2021	48	F	‒	2 months	‒	Mentum	Positive^a^	Yes	Yes	No	Minocycline 100 mg and topical tretinoin	Good response

ANA, antinuclear antibody; HN, homogeneous nuclear pattern; FSN, fine speckled nuclear pattern; CSN, coarse speckled nuclear pattern; SLE, systemic lupus erythematosus; DLE, discoid lupus erythematosus; F, female; M, male.

a Pattern not informed.

b Pattern and title not informed.

**Table 2:** Histopathological findings observed in the cases of the present series.

Histopathological findings	Case 1	Case 2	Case 3	Case 4	Case 5
Orthokeratosis	+	–	–	+	–
Epidermal thinning	+	+	+	+	–
Liquefaction degeneration	+	+	+	–	–
Lymphocytic infiltrate	+	+	+	+	+
Pigment incontinence	–	–	+	–	–
Follicular plug	+	+	+	+	+
Mucin deposition	+	+	–	–	–
Thickening of the basement membrane	–	+	–	+	+

## Discussion

CL is a rare presentation of CCLE, with only 17 cases described in the literature, to the best of the authors’ knowledge, and the present study has added five new cases. According to the literature, it predominates in women between the third and fourth decades of life, with a mean age of 38.9 years, different from the mean age of the cases in the present study, which was 56.6 years. Smoking, as in other forms of the disease, seems to be an important risk factor.

The pathogenesis of LC has not been well established yet. Follicular plugs, common in DLE, were observed in all cases in this series, as well as the presence of hyperkeratosis and inflammatory infiltrate near the pilosebaceous unit. These three findings, taken together, could justify its clinical expression with comedones, papules, and acneiform scars.^1^, ^5^, ^13^

The clinical manifestations include comedones, erythematous papules, and punctate scars affecting sun-exposed areas.^1^ The patients in this series had lesions on the face and on the auricular region. The presence of concomitant lesions of classic DLE, in addition to the confluence of lesions into infiltrated plaques, perilesional erythema, and telangiectasias, may be helpful in differentiating CL from other acneiform eruptions.^5^, ^9^ Pruritus is frequently described in the literature and was present in three of the five described patients.^1^, ^2^ Two patients developed inflammatory cysts. All patients had DLE lesions, concomitantly. The screening for antinuclear antibodies was positive in two cases, but only one of the patients was diagnosed with SLE.

Although the occurrence of acneiform lesions in areas that are photo-exposed and refractory to conventional treatment for acne may suggest CL, histopathology is crucial for the diagnosis.^1^, ^11^ The histological findings are similar to those seen in DLE, including hyperkeratosis, epidermal thinning, liquefaction degeneration of the basal layer, thickening of the basement membrane, pigment incontinence and predominantly lymphocytic inflammatory infiltrate in the papillary and periadnexal dermis; however, dilated follicular ostia, epidermal cysts, and prominent follicular plugs are patent in CL.^7^, ^13^ Of these classic findings, follicular plugs were the most prevalent, observed in all cases. The presence of mucin was observed in two cases, a finding not reported in previously published reports, but which is also seen in other subtypes of CCLE, especially in lupus tumidus. The increase in the number of cases of comedogenic lupus described may identify the true prevalence of this variant.

Differential diagnoses include acne vulgaris, comedogenic nevus, and nodular cutaneous elastoidosis with cysts and comedones (Favre-Racouchot disease).^5^, ^13^ As described in the literature, three cases had been previously diagnosed and treated as acne vulgaris, without success.

The treatment of this form of CCLE can be challenging, and photoprotection is essential in all cases, as recommended in the other variants. Topical therapy with retinoids such as tretinoin and tazarotene and the use of topical and/or intralesional corticosteroids may contribute to improvement, as seen in Case 5. However, most cases require systemic therapy,^7^, ^8^ with hydroxychloroquine (HCQ) being considered the first line of treatment.^3^ Two patients in this series had a partial response to methotrexate associated with HCQ. Only two patients showed complete symptom improvement, one after using methotrexate associated with dapsone (Case 1) and the other with topical corticosteroid associated with topical tretinoin (Case 5). Dapsone, successfully used in one of the cases in this series, even though its use has not been previously reported in CL, could have a potential role in the treatment, although further studies are needed.

## Conclusion

The present study contributes to the world literature by providing five new cases and intends to increase the understanding of this unusual and an understudied variant of CCLE. Due to its rarity and little knowledge of CL by dermatologists, the diagnosis can be delayed, with a negative impact on quality of life, since it is a dermatosis with the potential for significant unaesthetic complications, as also observed in classic DLE.^1^ CL should be included in the differential diagnosis of acneiform conditions in conjunction with atypical manifestations and poor response to the usual treatment.^9^ An early diagnosis and treatment could alleviate the morbidity and reduce the risk of scarring.^1^

## Financial support

None declared.

## Authors' contributions

Lucas Campos Garcia: Design and planning of the study; data collection; drafting and editing of the manuscript and critical review of relevant intellectual content; approval of the final version of the manuscript.

Isabela Boechat Morato: Design and planning of the study; data collection; drafting and editing of the manuscript; critical review of the intellectual content; approval of the final version of the manuscript.

Raquel Ferreira Queiroz de Melo: Drafting and editing of the manuscript and data collection; approval of the final version of the manuscript.

Everton Carlos Siviero do Vale: Design and planning of the study; drafting and editing of the manuscript and critical review of relevant intellectual content; approval of the final version of the manuscript.

## Conflicts of interest

None declared.
